# Clinical Evaluation of a Soap-Free Cleansing Lotion Containing Sodium Cocoyl Apple Amino Acids for Effective Impurity Removal and Skin Barrier Preservation in Healthy Adults With Diverse Skin Types

**DOI:** 10.7759/cureus.90538

**Published:** 2025-08-19

**Authors:** Maheshvari Patel, Ashik Khatri, Rajesh Khatri, Sheethal Sreevallabhan, Svenia Jose, Nayan Patel, Apeksha Merja

**Affiliations:** 1 Clinical Trials - Operations, NovoBliss Research Private Limited, Ahmedabad, IND; 2 Pharmacology, Swaminarayan University, Gandhinagar, IND; 3 Research and Development, Glowderma Lab Pvt. Ltd., Mumbai, IND; 4 Computer Science, University of Houston, Houston, USA; 5 Clinical Trials, NovoBliss Research Private Limited, Ahmedabad, IND

**Keywords:** cleansing, clean skin, hydration, porphyrin, skin barrier, skincare routines, skin impurities, sodium cocoyl apple amino acids

## Abstract

Background: Effective facial cleansing is essential for removing dirt, oil, and impurities without compromising the skin barrier and hydration. However, many conventional cleansers contain harsh surfactants that may strip the skin of its natural lipids, leading to dryness, irritation, and barrier disruption.

Aim: This study aimed to assess the safety and efficacy of a cleansing lotion containing cetyl alcohol and sodium cocoyl apple amino acids in eliminating skin surface impurities while preserving skin barrier function in healthy adult subjects with diverse skin types, including dry, oily, sensitive, combination, and normal.

Methods: This open-label, interventional study enrolled 27 participants, all of whom completed the 30-day study. The product’s effectiveness was assessed through key clinical and instrumental evaluations, focusing on skin surface impurities by assessing porphyrin levels (size, value, and quantity) and skin tone, along with hydration, barrier function, and overall dry skin score.

Results: After 30 days of use, significant improvements were observed across all parameters. Skin surface impurities reduced, as evidenced by porphyrin size, quantity, and values reduced to 0.29 ± 0.23, 7.11 ± 4.96, and 130.30 ± 16.28, respectively. Overall skin tone improved to 22.78 ± 14.58, skin hydration improved to 46.07 ± 8.47, and barrier function improved to 9.27 ± 1.83, with all changes being statistically significant (p < 0.0001). No adverse event was reported during the conduct of the study.

Conclusion: The soap-free cleansing lotion demonstrated clinical efficacy and excellent tolerability, effectively removing skin impurities while maintaining and enhancing skin hydration and maintaining the skin barrier function and overall skin appearance without causing irritation. These findings support its potential as a safe, evidence-based dermatological care, reinforcing its primary claims of effective cleansing and promoting visibly fresher and healthier skin.

## Introduction

Skin cleansing is a fundamental component of daily personal hygiene and skincare routines. The primary objective of cleansing products is to remove dirt, oil, and other impurities from the skin without compromising its natural barrier function [1]. However, it is essential that the formulation of these products is designed to minimize the risk of irritation, dryness, or other adverse dermatological effects, particularly with regular or prolonged use [2].

Dry and sensitive skin is a common but distinct condition often linked to dysfunction of the stratum corneum (SC), the outermost layer of the epidermis and key component of the skin barrier. This barrier is essential for retaining moisture by preventing transepidermal water loss (TEWL) and preserving the skin’s natural moisture balance [3,4]. It is essential to avoid further compromising the SC barrier during cleansing. Traditional skin cleansers frequently contain harsh surfactants such as sodium lauryl sulfate (SLS), which have been shown to compromise the integrity of the skin barrier by disrupting the SC and increasing TEWL [5]. Cleansers formulated with gentle synthetic surfactants and/or emollients that minimally disrupt the skin barrier are best suited for individuals with dry or sensitive skin [6].

Beyond maintaining hydration and skin barrier function, effective facial cleansers should also target surface impurities lodged within pores. The build-up of sebum, dead skin cells, and microbial byproducts can lead to clogged pores and contribute to acne. Elevated porphyrin levels are indicative of poor skin hygiene and pore blockage. Clinical evaluations have demonstrated that effective acne treatments, including cleansers, can significantly reduce porphyrin levels [7].

Test product is a soap-free dermatological formulation specifically developed to effectively remove surface impurities while preserving the integrity of the skin barrier and leaving the skin fresh and clean. It incorporates a combination of mild and natural surfactants, emollients, and humectants optimized for tolerability and hydration, particularly in subjects with sensitive, oily, or dry skin types. The formulation combines key ingredients, including sodium cocoyl apple amino acids, cetyl alcohol, caprylic/capric triglyceride, propylene glycol, aloe vera, and D-panthenol, that work synergistically to cleanse the skin, remove impurities, and maintain barrier integrity without causing irritation or dryness.

Sodium cocoyl apple amino acids are a gentle, biodegradable, and naturally derived surfactant from apple amino acids and coconut fatty acids. Known for their mild cleansing action, they effectively remove impurities without disrupting the skin barrier, making them ideal for all skin types, including sensitive and dry skin. They have demonstrated clinical efficacy in preserving skin hydration, supporting barrier integrity, and enhancing skin feel when compared to harsher surfactants such as sodium lauryl sulfate [1,8]. Cetyl alcohol is a long-chain fatty alcohol that functions as an emollient, emulsifier, and thickener in skincare formulations. It enhances product stability and texture while forming a protective lipid layer that helps reduce moisture loss without causing irritation or clogging pores [9,10]. Aloe vera is well known for its anti-inflammatory, moisturizing, and healing properties; it has demonstrated effectiveness in reducing skin irritation and enhancing barrier recovery [11].

Propylene glycol is used as a humectant, facilitating hydration by drawing moisture into the SC, and may enhance the penetration of active ingredients [12,13]. The formulation also includes D-panthenol (pro-vitamin B5), which improves hydration and promotes epithelial regeneration [14], and dimethicone, a silicone-based polymer known to reduce TEWL and improve skin texture without clogging pores [15]. Additionally, decyl glucoside, a non-ionic sugar-derived surfactant, is recognized for its mildness and compatibility with sensitive skin [16]. Importantly, it is preserved with phenoxyethanol, a widely accepted alternative to parabens known for its milder profile suitable for dry and sensitive skin [17].

This study assessed the safety and efficacy of soap-free cleansing lotion in healthy individuals with diverse skin types, including dry, oily, sensitive, combination, and normal skin. The primary objective was to evaluate the reduction in skin impurities and improvement in skin tone, while secondary outcomes assessed skin hydration, barrier integrity, dryness score, visual and tactile skin characteristics, and user satisfaction.

## Materials and methods

Ethical conduct of the study

This exploratory, prospective, open-label, interventional clinical study was conducted to evaluate the safety, in-use tolerability, and efficacy of the test product in healthy human subjects over a 30-day application period. The study was conducted in accordance with the ethical principles outlined in the Declaration of Helsinki, the International Council for Harmonisation (ICH) Good Clinical Practice (GCP) guidelines, and the ethical guidelines issued by the Indian Council of Medical Research (ICMR).

The study protocol received approval from the ACEAS Independent Ethics Committee on September 9, 2024. For the purpose of transparency and public accountability, the study was registered with the Clinical Trials Registry-India (CTRI/2024/10/075297; registered on 15/10/2024) and ClinicalTrials.gov (NCT06628258; registered on 02/10/2024).

All participants were enrolled after providing written informed consent, having been fully informed of the study’s objectives, methodology, potential risks and benefits, confidentiality safeguards, and their rights to voluntary participation and withdrawal without penalty. Participant safety, rights, and well-being were prioritized throughout the study, and compensation was provided at each scheduled visit in accordance with regulatory and ethical guidelines.

Study design

This safety and efficacy study was conducted at NovoBliss Research Private Limited, a contract research organization (CRO) center in Ahmedabad, India, over a usage period of 30 days. Subjects with varying skin types were enrolled, with recruitment starting on October 23, 2024, marking the first subject's visit, and concluding on December 4, 2024, with the final subject's visit. Primary endpoints included changes in skin impurities and skin tone, while secondary endpoints encompassed skin hydration, barrier function, overall dry skin (ODS) score, Physician Global Assessment (PGA) score, visual and tactile assessments, and a product perception questionnaire.

Instruments from Courage+Khazaka Electronic GmbH (Cologne, Germany), ISO 9001, 13485-certified bioinstrumentation, widely used in skin and hair research, including space-based studies, were selected for their precision and validated clinical use. These instruments provide accurate, standardized measurements, ensuring consistent and reliable assessment of skin and hair parameters (Tables 1, 2).

**Table 1:** Details of instruments used in the study. Devices from Courage+Khazaka were used for all measurements. UV-A: ultraviolet A; ITA: individual typology angle.

Instruments	Manufacturing details	Applications in the study
Visiopor® PP34N	Courage+Khazaka Electronic GmbH, Cologne, Germany (1.16.8)	To evaluate the severity of skin surface impurities by measuring porphyrin levels, indicated by their orange-red fluorescence under UV-A light. Severity of skin surface impurities
Colorimeter® CL 400		To evaluate skin color (L*, a*, b* & ITA values)
Corneometer® CM 825		To evaluate skin hydration
Tewameter^®^ TM Hex		To evaluate skin transepidermal water loss to see the skin barrier function

**Table 2:** Details of test product. Composition and usage instructions of the test product (Moiz Cleansing Lotion), based on product label and manufacturer information. EDTA: ethylenediaminetetraacetic acid.

Parameter	Test product
Test product	Moiz cleansing lotion contains cetyl alcohol, stearyl/alcohol, soap-free lotion base, sodium cocoyl apple amino acids, decyl glucoside, caprylic capric triglyceride, acrylates/C10-30 alkyl acrylate crosspolymer, propylene glycol, dimethicone, EDTA, triethanolamine, aloe vera, D-panthenol, phenoxyethanol, fragrance, and purified water
Storage condition	The test product was stored at the study site at room temperature, at 15°C to 30°C
Dosage form	Liquid
Mode of usage	Apply 5-10 mL of test product to wet skin. Massage gently for 1-2 minutes, then rinse and pat dry
Route of administration	Topical
Manufacturer details	
Manufactured by	Dermalogics
Marketed by	Glowderma Lab Private Limited

Eligibility criteria

Enrolled participants were healthy males and non-pregnant, non-lactating females, aged 18 to 65 years, with dry, oily, mixed, sensitive, or normal skin, as confirmed by dermatological assessment. Females of childbearing potential were required to have a negative pregnancy test and agree to use a reliable birth control method. Participants had no history of dermatological conditions, allergic reactions to cosmetics, or use of medications that could influence skin health, such as anti-inflammatories or corticosteroids. Individuals with severe acne, recent use of topical or systemic treatments, or any chronic illness affecting skin condition were excluded. Participants who committed to using only the test product, attending regular follow-ups, and providing written informed consent were included. Pregnant, breastfeeding, or individuals planning pregnancy during the study were not eligible.

Study population

A total of 27 healthy male and female subjects aged 18 to 51 years, representing diverse skin types, including dry, oily, mixed, normal, and sensitive, were enrolled in the study. The study population encompassed individuals from a wide range of occupational and lifestyle settings, reflecting the intended all-purpose use of the test product. Participants continued their regular daily activities without restrictions, allowing evaluation of the product under varied real-world conditions, including differences in sun exposure, environmental pollutants, and temperature variations. This approach ensured that the tolerability and efficacy of the cleansing lotion could be assessed across multiple practical use scenarios relevant to both indoor and outdoor users.

Study procedures and visits

The study was carried out at NovoBliss Research Private Ltd., Ahmedabad, over a 30-day usage period, which included three scheduled assessment visits. Subjects were pre-screened by the screening department and contacted telephonically prior to their enrolment visit. Female participants were instructed not to wear any facial makeup on assessment days. During visit 1 (day one), subjects underwent screening, enrolment, baseline assessments, and supervised on-site application of the test product, followed by immediate post-application evaluation. Visit 2 (day 15 ± 2 days) included mid-study follow-up and assessment of product use. Visit 3 (day 30 ± 2 days) marked the final evaluation and end of the study.

Statistical analysis

Continuous variables were summarized using descriptive statistics, including sample size (N), mean, standard deviation (SD), median, minimum, and maximum values. Categorical variables were presented as frequencies and percentages, with graphical representations provided where applicable. For quantitative variables, data were compared from baseline to post usage of the product using the paired t-test or the Wilcoxon signed-rank test. Statistical analysis was conducted using IBM SPSS Statistics for Windows version 29.0.1.0 (IBM Corp., Armonk, NY) and Microsoft Excel 2019 (Microsoft Corporation, Redmond, WA), applying a 5% level of significance, and all 27 subjects completed the study.

Sample size calculation

The sample size was calculated using R software (R Foundation for Statistical Computing, Vienna, Austria) based on the primary endpoint of TEWL (g/h/m²), measured from baseline to four weeks after applying the test treatment. The mean and standard deviation (SD) of TEWL at baseline and at week four were estimated as 11.48 (SD: ±2.38) and 12.45 (SD: ±2.05), respectively. The assumed mean change and common SD for the calculation were 0.97 (SD: ±1.90), derived from reference literature relevant to the current study design. The null hypothesis tested was that there would be no change in TEWL after four weeks of product use, while the alternative hypothesis posited a significant change. A paired comparison approach was used to evaluate TEWL before and after treatment. To detect a true mean change with 80% statistical power at a one-sided 5% significance level, the calculated effect size was 0.5105, resulting in a minimum required sample size of 25 subjects. To account for an anticipated 15% dropout rate, the total sample size was adjusted to 30 subjects to ensure that at least 25 participants would complete the study.

Based on the sample size and clinical safety, efficacy, and in-use tolerability study, 27 subjects of either gender aged between 18 and 65 years old (both inclusive) at the time of consent were enrolled, and all 27 subjects completed the study [18].

## Results

The study comprised a total of 27 participants, including 14 females and 13 males, with a mean age of 35.37 years. Detailed summary of the participants’ demographic characteristics, including gender distribution, predominant race, age, body weight, and height, is shown in Table 3. A total of 27 subjects were enrolled, and a total of 27 subjects completed the study.

**Table 3:** Subject demographics and baseline characteristics. Data derived from the current study.

Parameter	Statistic	Total enrolled subjects (N = 27)
Gender, n (%)	Female	14 (51.85%)
	Male	13 (48.15%)
Race, n (%)	Asian	27 (100%)
ConMed history, n (%)	No	27 (100.00%)
Age (year)	Mean (SD)	35.37 (10.66)
	Median	35
	Min, Max	18.00, 51.00
Weight (kg)	Mean (SD)	66.33 (15.71)
	Median	64.70
	Min, Max	37.30, 98.42
Height (cm)	Mean (SD)	162.09 (9.68)
	Median	163.00
	Min, Max	146.00, 179.00

Assessment of skin impurities (porphyrin size, quantity, and values) using Visiopor® PP 34N

Porphyrins are metabolic byproducts of skin-resident bacteria and serve as markers of surface-level skin impurities. A reduction in porphyrin levels indicates a corresponding decrease in surface impurities, reflecting improved skin cleanliness [7].

Porphyrin size using the Visiopor® PP 34N (Courage+Khazaka Electronic GmbH, Cologne, Germany) showed significant reductions over time. The initial porphyrin size measured 1.99 ± 1.27, decreasing to 1.19 ± 0.86 (40.96% reduction, p-value < 0.0001) 20 minutes after applying the test product, further reduced to 0.76 ± 0.55 (60.82% reduction, p-value < 0.0001) by day 15, and to 0.29 ± 0.23 (85.76% reduction, p-value < 0.0001) by day 30.

Additionally, the porphyrin quantity initially was 26.41 ± 12.17, reduced to 19.67 ± 10.02 (25.75% reduction, p-value < 0.05) after 20 minutes, further to 13.78 ± 8.39 (49.42% reduction, p-value < 0.05) by day 15, and 7.11 ± 4.96 (74.22% reduction, p-value < 0.05) by day 30.

The porphyrin value began at 155.48 ± 18.10 and dropped to 146.44 ± 18.73 (5.82% reduction, p-value <0.05) after 20 minutes, with a further decrease to 139.59 ± 18.34 (10.15% reduction, p-value < 0.05) by day 15, and 130.30 ± 16.28 (15.99% reduction, p-value < 0.05) by day 30.

**Figure 1:**
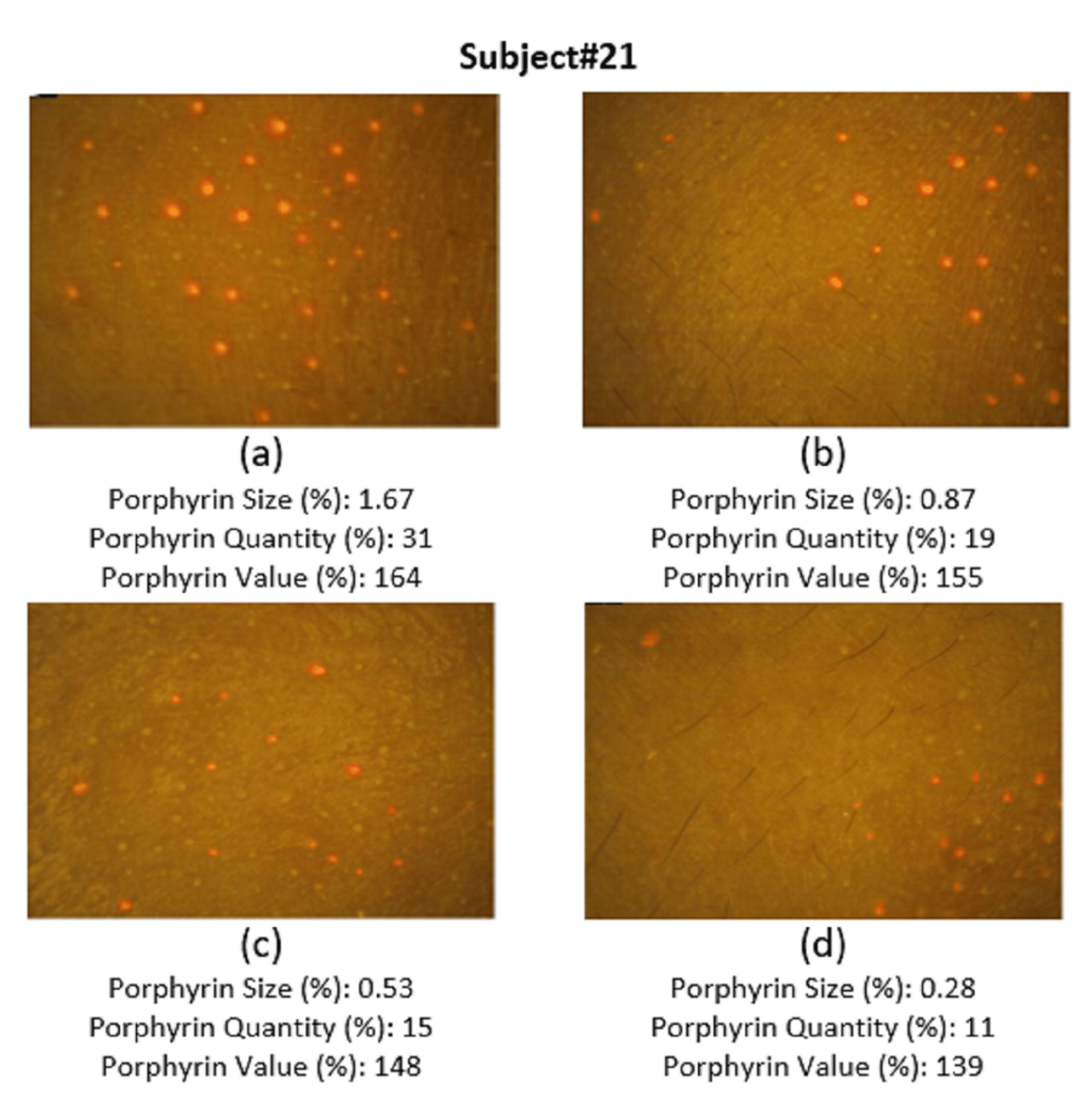
Assessment of skin impurities (a) at baseline, (b) after 20 minutes of product application, (c) on day 15, and (d) on day 30.

As reflected in the photographs, porphyrin levels were elevated at baseline and markedly decreased at subsequent visits following application of the cleansing lotion (Figure 1).

Assessment of skin color (change in L*, a*, b* and ITA angle values)

The L* value, which indicates skin brightness, showed a statistically significant improvement. At baseline, the mean value was 55.59 ± 4.92, which increased to 56.66 ± 4.98 or 1.92% at 20 minutes post-application on day one, 57.33 ± 4.97 or 3.15% on day 15, and 58.06 ± 5.19 or 4.46% on day 30 (p-value < 0.0001).

The a* value, representing skin redness, demonstrated significant reductions. At baseline, the mean value was 11.77 ± 1.79, which reduced to 10.96 ± 1.93 or 6.47% at 20 minutes post-application on day one (p-value < 0.05), 10.04 ± 1.96 or 14.46% on day 15 (p-value < 0.0001), and 9.08 ± 2.08 or 22.89% on day 30 (p-value < 0.0001).

Similarly, the b* value reflects skin pigmentation. At baseline, the mean value was 19.41 ± 1.88, which reduced to 19.98 ± 1.97 or 3.09% at 20 minutes post-application on day one (p-value < 0.05), 19.12 ± 1.60 or 0.89% on day 15 (p-value < 0.5), and 17.95 ± 1.93 or 6.69% on day 30, all statistically significant (p-value < 0.05).

**Figure 2:**
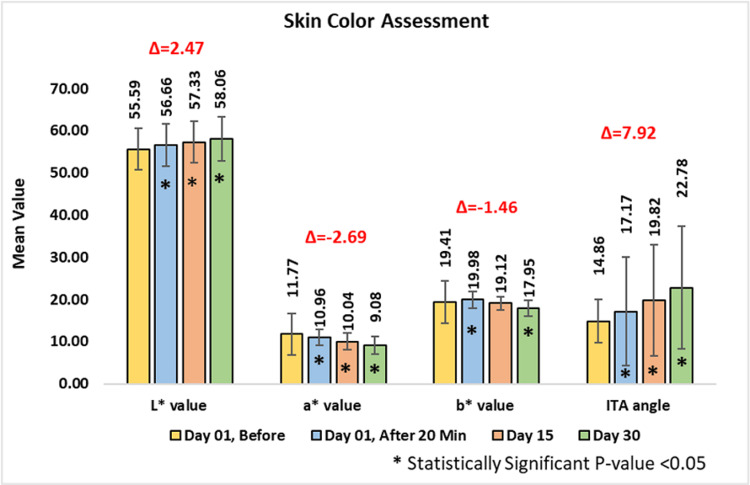
Skin color assessment. Paired t-test was used for statistical analysis. ITA: individual typology angle.

The individual typology angle (ITA), an indicator of overall skin tone, improved significantly. At baseline, the mean value was 14.86 ± 13.09, which improved to 17.17 ± 12.85 or 43.69% at 20 minutes post-application on day one, 19.82 ± 13.15 or 96.76% on day 15, and 22.78 ± 14.58 or 118.52% on day 30 (p-value < 0.0001) (Figure 2).

Secondary efficacy endpoints

Assessment of Skin Hydration Using Corneometer® CM 825

**Figure 3:**
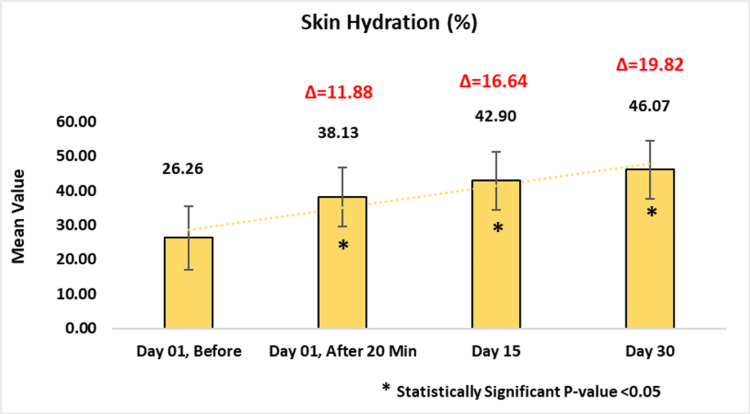
Assessment of skin hydration. Paired t-test was used for statistical analysis.

Skin hydration, evaluated using the Corneometer® CM 825 (Courage+Khazaka Electronic GmbH, Cologne, Germany), showed significant and clinical improvements. Initially, it measured 26.26 ± 9.15, which increased to 38.13 ± 8.44 or 55.71% (p-value < 0.0001) after 20 minutes of applying the test product. Subsequent increases from baseline in skin hydration were observed as 42.90 ± 8.42 or 77.04% (p-value < 0.0001) at day 15 and 46.07 ± 8.47 or 90.98% at day 30 (p-value < 0.0001) (Figure 3).

**Figure 4:**
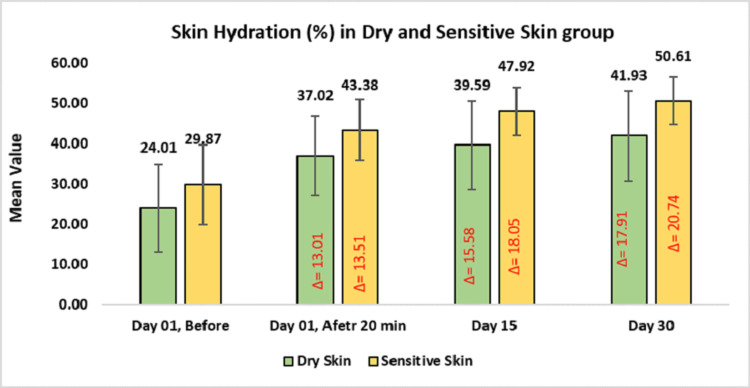
Assessment of skin hydration in dry and sensitive skin group. Paired t-test was used for statistical analysis.

Subgroup analysis demonstrated a notable improvement in skin hydration among participants with dry and sensitive skin. For the dry skin group, hydration increased from baseline by 37.02 ± 9.83 or 75.28% (p-value < 0.0001) on day one after 20 minutes of applying the test product, 39.59 ± 10.94 on day 15 or 87.39% (p-value < 0.0001), and 41.93 ± 11.21 or 97.71% on day 30. In the sensitive skin group, it reduced to 43.38 ± 7.47 or 51.94% (p-value < 0.0001) on day one after 20 minutes of applying the test product, 47.92 ± 5.95 on day 15 or 70.64% (p-value < 0.0001), and 50.61 ± 5.75 or 79.86% on day 30 (Figure 4).

Assessment of Skin Barrier Function Using TEWAMeter® TM Hex

**Figure 5:**
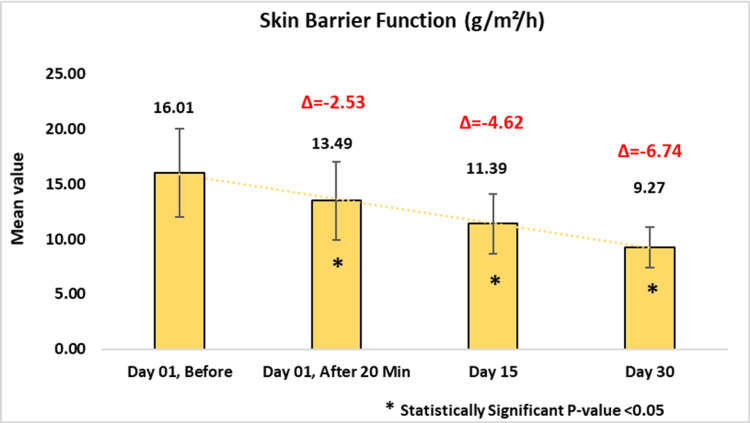
Assessment of skin barrier function. Paired t-test was used for statistical analysis.

Compared to baseline, the use of the test product led to progressive improvement in skin barrier function, as evidenced by a reduction in TEWL. Initially, it measured 16.01 ± 4.03, which reduced to 13.49 ± 3.56 or 15.65% (p-value < 0.0001) on day one after 20 minutes of applying the test product, 11.39 ± 2.70 or 28.19% on day 15 (p-value < 0.0001) and 9.27 ± 1.83 or 40.73% on day 30 (p-value < 0.0001) (Figure 5).

**Figure 6:**
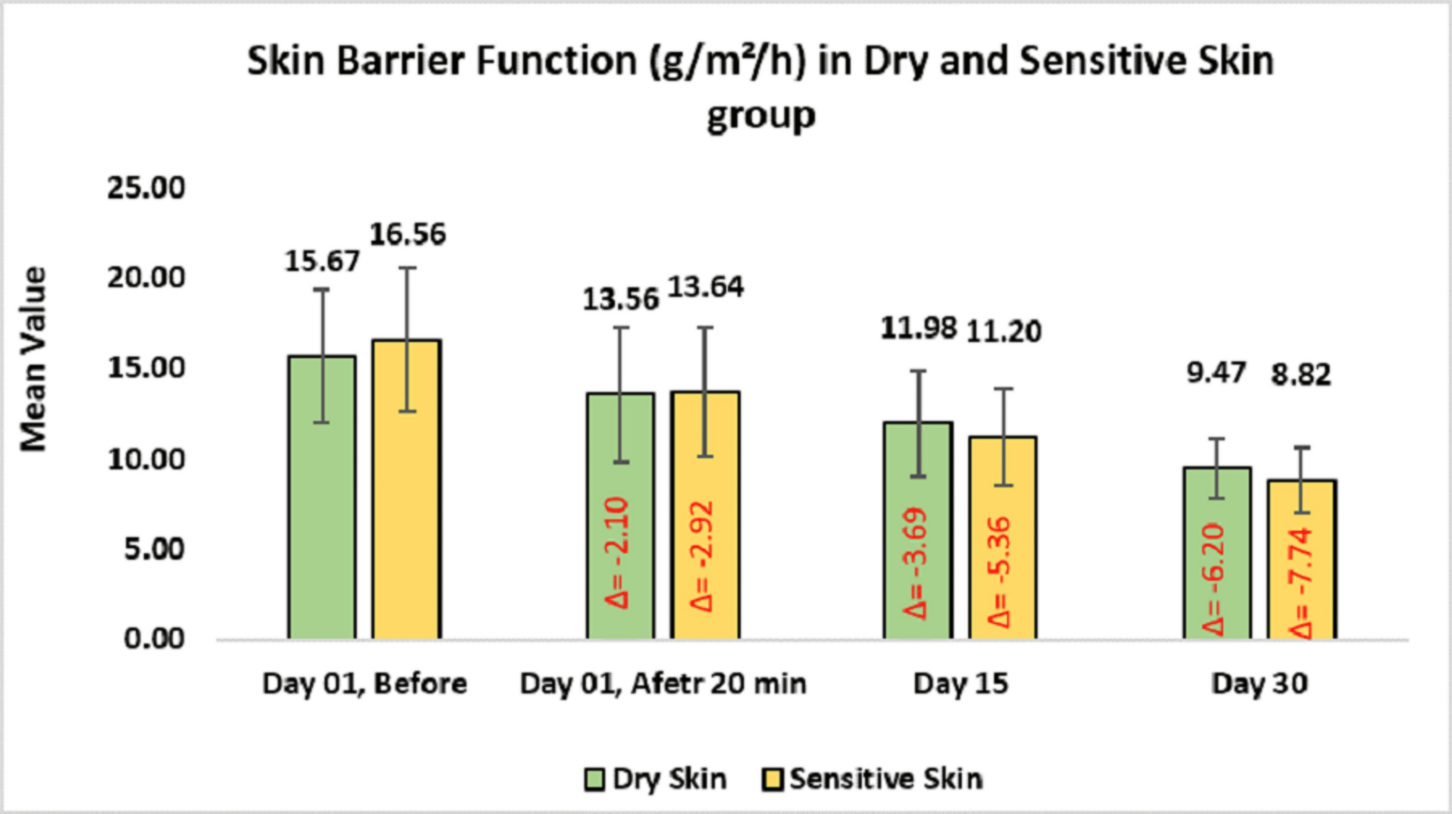
Assessment of skin barrier function in dry and sensitive skin group. Paired t-test was used for statistical analysis.

Subgroup analysis demonstrated a notable reduction in TEWL among participants with dry and sensitive skin. For the dry skin group, TEWL decreased from baseline by 13.56 ± 3.73 or 15.65% (p-value < 0.0001) on day one after 20 minutes of applying the test product, 11.98 ± 2.94 or 23.60% on day 15 (p-value < 0.0001) and 9.47 ± 1.66 or 38.61% on day 30. In the sensitive skin group, it reduced to 13.64 ± 3.57 or 17.88% (p-value < 0.0001) on day one after 20 minutes of applying the test product, 11.20 ± 2.68 or 32.29% on day 15 (p-value < 0.0001), and 8.82 ± 1.77 or 46.13% on day 30 (Figure 6).

Assessment Overall Dry Skin Score - Dermatological Assessment

**Figure 7:**
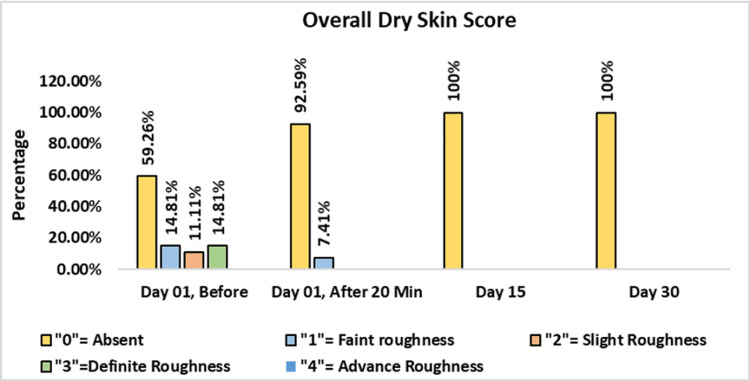
Assessment of overall dry skin score. Paired t-test was used for statistical analysis.

At baseline, 59.26% of subjects exhibited no skin dryness, while 14.81% had faint scaling, 11.11% presented with small scales along with larger scales, slight roughness, and whitish appearance, and 14.81% showed small and larger scales uniformly distributed. Following the application of the test product, 92.59% of subjects demonstrated no skin dryness, and 7.41% exhibited only faint scaling at 20 minutes post-application. By day 15 and day 30, 100% of the subjects experienced a complete absence of skin dryness. These results conclusively highlight the efficacy of the test product, a cleansing lotion, in addressing and eliminating skin dryness over time with consistent use (Figure 7).

*Physician Global Assessment (PGA* *) - Dermatological Evaluations*

**Figure 8:**
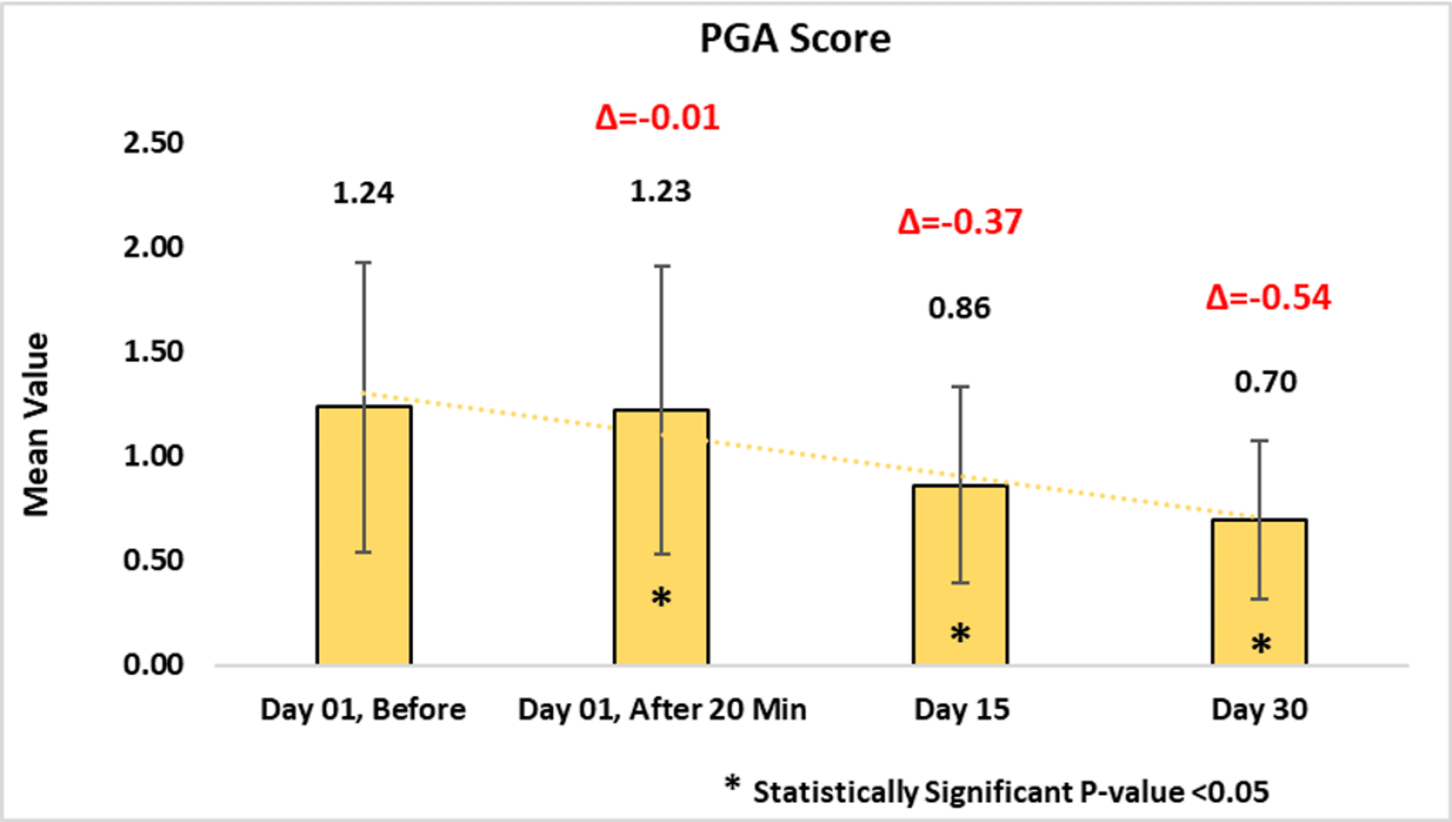
Assessment of Physician Global Assessment (PGA) score. Paired t-test was used for statistical analysis.

In the PGA scale assessment, before application of the test product, 1.23 ± 0.69, 0.86 ± 0.47, and 0.70 ± 0.38 reductions were observed with p-value < 0.05 on day zero post 20 minutes, day 15, and day 30. In the X time measurement, up to 2x reduction was observed post 30 days of the product usage (Figure 8).

Change in Visual & Tactile Assessment by Scoring Scale: Dermatological Assessment

Improvements in skin condition were observed across all parameters following the application of the test product. At baseline, a proportion of subjects exhibited skin scaliness (29.63%), redness (18.52%), dryness (40.74%), roughness (51.85%), and varying degrees of skin texture irregularities. Notably, by day 30, 100% of participants showed complete resolution of scaliness, dryness, roughness, and redness, with a statistically significant reduction in scaliness (p < 0.05). No cases of itchiness were reported throughout the study period. Skin smoothness progressively improved, with 55.56% of participants exhibiting “very smooth” skin by day 30 compared to none at baseline. These findings suggest that regular use of the test product effectively improves skin texture, hydration, and tolerability in a diverse population (Table 4).

**Table 4:** Visual and tactile assessment. Data derived from the current study. Scaliness, roughness, and redness: 0 = absent, 1 = slight, 2 = moderate, 3 = severe, 4 = extreme. Itchiness: 0 = none, 1 = mild, 2 = moderate, 3 = severe. Dryness: 0 = no skin dryness, 1 = slight xerosis, 2 = moderate xerosis, 3 = severe xerosis, 4 = extreme xerosis. Smoothness: 0 = very rough, 1 = rough, 2 = slightly rough.

Parameter	Score	Visit 1 (before test product usage)	Visit 1 (T20 min) (after test product usage)	Visit 2 (day 15)	Visit 3 (day 30)
Skin scaliness	"0"	19 (70.37%)	27 (100%)	27 (100%)	27 (100%)
	"1"	5 (18.52%)	0 (0.00%)	0 (0.00%)	0 (0.00%)
	"2"	3 (11.11%)	0 (0.00%)	0 (0.00%)	0 (0.00%)
	"3"	0 (0.00%)	0 (0.00%)	0 (0.00%)	0 (0.00%)
	"4"	0 (0.00%)	0 (0.00%)	0 (0.00%)	0 (0.00%)
Skin redness	"0"	22 (81.48%)	27 (100%)	27 (100%)	27 (100%)
	"1"	4 (14.81%)	0 (0.00%)	0 (0.00%)	0 (0.00%)
	"2"	1 (3.7%)	0 (0.00%)	0 (0.00%)	0 (0.00%)
	"3"	0 (0.00%)	0 (0.00%)	0 (0.00%)	0 (0.00%)
	"4"	0 (0.00%)	0 (0.00%)	0 (0.00%)	0 (0.00%)
Skin dryness	"0"	16 (59.26%)	24 (88.89%)	27 (100%)	27 (100%)
	"1"	2 (7.41%)	3 (11.11%)	0 (0.00%)	0 (0.00%)
	"2"	6 (22.22%)	0 (0.00%)	0 (0.00%)	0 (0.00%)
	"3"	3 (11.11%)	0 (0.00%)	0 (0.00%)	0 (0.00%)
	"4"	0 (0.00%)	0 (0.00%)	0 (0.00%)	0 (0.00%)
Skin roughness	"0"	13 (48.15%)	27 (100%)	27 (100%)	27 (100%)
	"1"	8 (29.63%)	0 (0.00%)	0 (0.00%)	0 (0.00%)
	"2"	6 (22.22%)	0 (0.00%)	0 (0.00%)	0 (0.00%)
	"3"	0 (0.00%)	0 (0.00%)	0 (0.00%)	0 (0.00%)
	"4"	0 (0.00%)	0 (0.00%)	0 (0.00%)	0 (0.00%)
Skin itchiness	"0"	27 (100%)	27 (100%)	27 (100%)	27 (100%)
	"1"	0 (0.00%)	0 (0.00%)	0 (0.00%)	0 (0.00%)
	"2"	0 (0.00%)	0 (0.00%)	0 (0.00%)	0 (0.00%)
	"3"	0 (0.00%)	0 (0.00%)	0 (0.00%)	0 (0.00%)
	"4"	0 (0.00%)	0 (0.00%)	0 (0.00%)	0 (0.00%)
Skin smoothness	"0"	15 (55.56%)	3 (11.11%)	0 (0.00%)	0 (0.00%)
	"1"	9 (33.33%)	5 (18.52%)	1 (3.70%)	0 (0.00%)
	"2"	3 (11.11%)	7 (25.93%)	4 (14.81%)	3 (11.11%)
	"3"	0 (0.00%)	9 (33.33%)	11 (40.74%)	9 (33.33%)
	"4"	0 (0.00%)	3 (11.11%)	11 (40.74%)	15 (55.56%)

Effect of the Test Product in Terms of Product Perception

Based on the product perception questionnaire administered after 30 days of use, 100% of subjects expressed high satisfaction (rated on a 1-9 scale) across all evaluated parameters. Participants consistently reported effective removal of dirt and significant improvements in skin hydration, smoothness, and reduction of dryness, roughness, redness, scaliness, itchiness, and oiliness. Additionally, all subjects endorsed the test product for providing instant hydration and indicated overall satisfaction with its performance, demonstrating excellent user acceptance and tolerability across diverse skin types.

## Discussion

This study evaluated the safety and efficacy of soap-free cleansing lotion, primarily targeting the reduction of skin impurities, enhancement of skin tone, hydration, and reinforcement of barrier integrity. These outcomes were measured both clinically and instrumentally at baseline and following product application to determine its overall effectiveness.

A significant reduction in porphyrin-related skin impurities was observed using Visiopor® PP 34N analysis. Porphyrin size, quantity, and intensity values decreased notably over the 30-day period (p < 0.05), reflecting a strong cleansing effect and improved skin clarity. Numerous studies have shown that a decrease in porphyrins, as measured by Visiopor®, is associated with the removal of impurities that contribute to acne [19,20].

Improvements in skin brightness, tone uniformity, and reduction of pigmentation were evident through consistent changes in L*, a*, b*, and ITA values. Over the 30-day usage period, the product significantly enhanced skin brightness (L* values) and uniformity of skin tone (ITA angle), while reducing redness (a* values) and pigmentation (b* values) with a statistically significant p-value < 0.05.

Secondary efficacy outcomes further supported the formulation’s skin benefits. Corneometric analysis revealed significant and sustained improvements in skin hydration beginning as early as 20 minutes post-application and continuing through day 30. This hydration effect aligns with the observed enhancements in skin barrier function, as measured by reduced TEWL via the TEWAMeter® TM Hex. These results underscore the moisturizing properties of the product and its role in reinforcing the skin’s protective barrier. Additionally, subgroup analysis confirmed that the test cleansing lotion is effective and well-suited for individuals with dry and sensitive skin. In the dry skin group, TEWL decreased by 38.61% by day 30, while hydration increased by 97.71% (all p < 0.0001). Similarly, the sensitive skin group showed TEWL reductions of 46.13%, with hydration improving by 79.86% over the same time points (all p < 0.0001). These results highlight the product’s ability to restore moisture and strengthen the skin barrier in dryness-prone and sensitive skin types.

Moreover, the PGA scale assessment demonstrated statistically significant reductions in skin condition scores over time by day 30. Visual and tactile scoring further reinforced these findings, highlighting the product’s ability to improve both the appearance and feel of the skin. Notably, no participants reported itchiness, indicating high tolerability and suitability for sensitive skin types.

Subjective feedback from participants was overwhelmingly positive. All users reported high satisfaction and perceived improvements in hydration, skin smoothness, and cleanliness, as well as a notable reduction in other textural irregularities. The 100% user endorsement reflects strong product acceptance and suggests that the formulation is well-suited for regular use across a diverse demographic.

Recent studies explored that wash gel containing dexpanthenol, which is also present in the test product, resulted in 58.8% improvement in skin hydration compared to baseline (p < 0.001) after 14 days of application [19]. In comparison, the test product achieved similar results with a 77.04% (p < 0.001) improvement after 15 days. A study involving 30 individuals with sensitive or problematic skin demonstrated that a soap-free, mildly acidic cleansing emulsion caused only mild disruption to the epidermal barrier, comparable to washing with water alone. Despite a slight increase in pH and minor dehydration, no significant impairment of skin function was observed over 21 days [21]. These findings support the use of gentle, soap-free formulations like the test product in maintaining skin barrier integrity during routine cleansing.

While the study did not include a control group, the consistent and statistically significant improvements observed across multiple clinical and instrumental parameters strongly support the efficacy of the test product. The open-label design reflects real-world use conditions and provides valuable insights into product performance across diverse skin types. However, incorporating a control or comparator arm in future studies would further strengthen the evidence by isolating the product’s effects and minimizing the influence of external variables. Such an approach would enhance the robustness and generalizability of the findings.

The study was conducted with a relatively small sample size (n = 27), lacked a control group, and followed an open-label design over a short duration, it nevertheless offers meaningful preliminary evidence supporting the product’s potential benefits. These findings lay the groundwork for future large-scale, controlled studies to further validate the outcomes.

## Conclusions

The tested soap-free cleansing lotion demonstrated a favorable safety and efficacy profile in healthy adults with a range of skin types, including sensitive skin. Unlike traditional foaming cleansers that can compromise the skin barrier, this lotion-based, soap-free formulation uses naturally derived surfactants to gently and effectively remove surface impurities while preserving skin barrier integrity. Enriched with skin-compatible ingredients such as cetyl alcohol, stearyl alcohol, sodium cocoyl apple amino acids, aloe vera, and phenoxyethanol, the cleanser delivers a synergistic blend of long-lasting hydration, nourishment, and soothing benefits. Clinical evaluations showed significant improvements in skin hydration, texture, and smoothness, along with notable reductions in porphyrin levels and TEWL, indicating effective cleansing and support for the skin’s natural barrier.

These findings demonstrate the product’s advantage over conventional cleansers, offering a milder, well-tolerated alternative that effectively maintains skin health without inducing irritation or dryness. Its balanced, soap-free formulation is particularly suitable for daily use for all skin types, supporting skin hygiene and hydration. The cleanser presents a clinically relevant option for routine dermatological care and evidence-based skincare practices.

## References

[1] Güder S Sr, Güder H (2023). Investigation of the chemical content and user comments on facial cleansing products. Cureus.

[2] Mukhopadhyay P (2011). Cleansers and their role in various dermatological disorders. Indian J Dermatol.

[3] Rawlings AV, Harding CR (2004). Moisturization and skin barrier function. Dermatol Ther.

[4] Baker P, Huang C, Radi R, Moll SB, Jules E, Arbiser JL (2023). Skin barrier function: the interplay of physical, chemical, and immunologic properties. Cells.

[5] Ananthapadmanabhan KP, Moore DJ, Subramanyan K, Misra M, Meyer F (2004). Cleansing without compromise: the impact of cleansers on the skin barrier and the technology of mild cleansing. Dermatol Ther.

[6] Subramanyan K (2004). Role of mild cleansing in the management of patient skin. Dermatol Ther.

[7] Patel MN, Patel NK, Merja AM, Patnaik S (2024). Clinical evaluation of the efficacy, safety, and in-use tolerability of a Diacnemide™-containing acne kit (facial serum and cleanser) regimen for the synergistic management of facial acne in adults. Cureus.

[8] Fainerman VB, Möbius D, Miller R (2001). Surfactants: Chemistry, Interfacial Properties, Applications. https://www.sciencedirect.com/bookseries/studies-in-interface-science/vol/13/suppl/C.

[9] Mohiuddin AK (2019). Skin care creams: formulation and use. OSP J Clin Trials.

[10] Boyer IJ, Bergfeld WF, Heldreth B, Fiume MM, Gill LJ (2017). The Cosmetic Ingredient Review Program-expert safety assessments of cosmetic ingredients in an open forum. Int J Toxicol.

[11] Kumari P, Bhatt DK, Manoharan K (2023). Exploring clinical effects and usage patterns of a daily face cleanser enriched with glycolic acid, aloe vera, and vitamin-E for acne management: a post-hoc analysis. Int J Res Dermatol.

[12] Pemberton MA, Kimber I (2023). Propylene glycol, skin sensitisation and allergic contact dermatitis: a scientific and regulatory conundrum. Regul Toxicol Pharmacol.

[13] Warshaw EM, Belsito DV, Taylor JS (2013). North American Contact Dermatitis Group patch test results: 2009 to 2010. Dermatitis.

[14] Camargo FB Jr, Gaspar LR, Maia Campos PM (2011). Skin moisturizing effects of panthenol-based formulations. J Cosmet Sci.

[15] Pawar BA, Falk B (2021). Use of advanced silicone materials in long-lasting cosmetics. Surface Science and Adhesion in Cosmetics.

[16] Fiume MM, Heldreth B, Bergfeld WF (2013). Safety assessment of decyl glucoside and other alkyl glucosides as used in cosmetics. Int J Toxicol.

[17] Ma X, Wang H, Song Y, Pan Y (2021). Skin irritation potential of cosmetic preservatives: an exposure-relevant study. J Cosmet Dermatol.

[18] (2025). National Eczema Association. https://nationaleczema.org/.

[19] Perugini P, Grignani C, Condrò G (2023). Skin microbiota: setting up a protocol to evaluate a correlation between the microbial flora and skin parameters. Biomedicines.

[20] Peltier E, Trapp S, de Salvo R (2022). A new dexpanthenol-containing liquid cleanser for atopic-prone skin: Results from two prospective clinical studies evaluating cutaneous tolerability, moisturization potential, and effects on barrier function. J Cosmet Dermatol.

[21] Bornkessel A, Flach M, Arens-Corell M, Elsner P, Fluhr JW (2005). Functional assessment of a washing emulsion for sensitive skin: mild impairment of stratum corneum hydration, pH, barrier function, lipid content, integrity and cohesion in a controlled washing test. Skin Res Technol.

